# The Impact of Glycerol on an Affibody Conformation and Its Correlation to Chemical Degradation

**DOI:** 10.3390/pharmaceutics13111853

**Published:** 2021-11-03

**Authors:** Ingrid Ramm, Adrian Sanchez-Fernandez, Jaeyeong Choi, Christian Lang, Jonas Fransson, Herje Schagerlöf, Marie Wahlgren, Lars Nilsson

**Affiliations:** 1 Department of Food Technology, Engineering and Nutrition, Lund University, 221 00 Lund, Sweden; adrian.sanchez-fernandez@food.lth.se (A.S.-F.); feelcjy@gmail.com (J.C.); marie.wahlgren@food.lth.se (M.W.); lars.nilsson@food.lth.se (L.N.); 2 Jülich Centre for Neutron Science, Heinz Maier-Leibnitz Zentrum, 85748 Garching, Germany; c.lang@vta.cc; 3 SOBI, Tomtebodavägen 23A, 171 65 Stockholm, Sweden; 4 Department of Chemical Engineering, Lund University, 221 00 Lund, Sweden; herje.schagerlof@chemeng.lth.se

**Keywords:** pharmaceutical proteins, liquid formulation, glycerol, protein stability, chemical degradation, chemical stability, protein conformation

## Abstract

The addition of glycerol to protein solutions is often used to hinder the aggregation and denaturation of proteins. However, it is not a generalised practice against chemical degradation reactions. The chemical degradation of proteins, such as deamidation and isomerisation, is an important deteriorative mechanism that leads to a loss of functionality of pharmaceutical proteins. Here, the influence of glycerol on the chemical degradation of a protein and its correlation to glycerol-induced conformational changes is presented. The time-dependent chemical degradation of a pharmaceutical protein, GA-Z, in the absence and presence of glycerol was investigated in a stability study. The effect of glycerol on protein conformation and oligomerisation was characterised using asymmetric field-flow fractionation and small-angle neutron scattering in a wide glycerol concentration range of 0–90% *v*/*v*. The results from the stability study were connected to the observed glycerol-induced conformational changes in the protein. A correlation between protein conformation and the protective effect of glycerol against the degradation reactions deamidation, isomerisation, and hydrolysis was found. The study reveals that glycerol induces conformational changes of the protein, which favour a more compact and chemically stable state. It is also shown that the conformation can be changed by other system properties, e.g., protein concentration, leading to increased chemical stability.

## 1. Introduction

Proteins are increasingly becoming a key substance in the modern high-tech industry both as a functional end-product and as part of sustainable green production. Areas in which proteins are utilised include therapeutic proteins, protein-based assays, and technical enzymes, which play an important role within green chemistry. The chemical degradation of proteins is an important deteriorative mechanism that leads to a loss of functionality of proteins. In solution, proteins can be chemically degraded by several reaction routes, e.g., oxidation, reduction, deamidation, isomerisation, and hydrolysis [1]. Deamidation and isomerisation are two of the most common chemical degradation reactions [2,3], and may cause a loss of protein function [2,4,5]. For therapeutic proteins, these two reactions may not only affect drug efficacy [4,6] but also induce physical degradation as aggregation [5,7,8] and increase the risk of immunogenicity in patients [2,3,4]. Therefore, understanding the chemical degradation pathways of proteins and elucidating how to suppress them becomes an important step in drug development.

Due to its importance, a significant amount of work has been performed to understand the chemical degradation reactions of proteins. The rate of deamidation of asparagine and isomerisation of aspartic acid has been shown to depend on the physical properties of the surrounding solution and amino acid residues [9,10,11,12]. Deamidation and isomerisation start with an attack from a nucleophilic, deprotonated nitrogen in the peptide backbone against the amide centre in the side chains of asparagine and aspartic acid [10]. In the 1980s, Clarke showed that there is a critical distance between the nucleophilic nitrogen and the amide centre in the protein structure (Clarke’s Conjecture) above which the reactions will not occur [9]. Hence, protein conformations leading to a shorter distance between the reaction sites favour deamidation and isomerisation [9]. Higher mobility of the peptide backbone increases the risk for conformations where deamidation and isomerisation would be more likely to occur. The primary, secondary, and tertiary protein structures all affect the intra-molecular mobility and distances of a protein, and therefore influence the rate of deamidation and isomerisation [9,11,13,14,15]. There are, however, no extensive studies examining how quaternary structures of proteins, such as oligomerisation, affect deamidation or other chemical degradation reactions. If oligomerisation leads to a decrease in peptide backbone mobility, deamidation and isomerisation could be inhibited. In a study by Xie et al. [14], it was hypothesised that the formation of trimers may protect the investigated protein against deamidation; however, the authors conclude that if that was the case, the protective effect of the secondary and tertiary structures was more prominent than the changes in protein self-association.

A common approach to enhancing the physical stability of proteins is to add polyols as cosolvents e.g., glycerol [12,16,17], which stabilises the native conformation of proteins against aggregation and denaturation [18,19]. It has also been shown that glycerol can stabilise the activity of enzymes [20] and prevent the precipitation of insulin in infusion pumps [21]. However, there are, to the best of our knowledge, no studies investigating how glycerol affects chemical degradation reactions, such as the deamidation and isomerisation of proteins. For peptides, previous studies have shown that glycerol can decrease deamidation and isomerisation rates in short peptides containing six amino acids [6,10]. This result could potentially be extended to the protection of proteins, but it must be kept in mind that proteins, unlike short peptides, form defined folded structures, which are known to affect chemical degradation reactions. The decrease in aggregation and denaturation of native proteins caused by glycerol is believed to be the result of a shift of the protein conformation towards more compact and ordered states [16]. Several studies have shown that glycerol reduces the apparent partial specific protein volume and increases the apparent adiabatic compressibility of proteins [18,19,22,23]. It has also been shown that glycerol decreases the flexibility of hen egg-white lysozyme by lowering the amount of unstructured protein [24]. The preferential interaction theory is commonly applied to explain the increased physical stability induced by glycerol [25]. The theory describes how co-solvent molecules either preferentially interact with or are excluded from the surface of proteins. The exclusion of glycerol from the surface of proteins has been shown several times in experimental studies and in simulations applying the preferential interaction theory [12,16,18,26,27,28].

Despite the great importance of inhibiting chemical degradation and the prospects of using glycerol to lower the rate of these reactions, the understanding of glycerol stabilisation against the chemical degradation reactions of proteins has not been extensively investigated. The aim of this investigation is to understand the influence of glycerol on the chemical degradation reactions of proteins and its correlation to glycerol-induced conformational changes. A small pharmaceutical protein, GA-Z, was investigated under different formulation conditions in the presence and absence of glycerol. GA-Z is an affibody, which is a class of engineered proteins of increasing interest in biotechnological and medical applications [29,30]. The protein consists of two domains, a Z-domain and an albumin binding domain, covalently connected by a linker. GA-Z has been shown to chemically degrade in an aqueous solution, mainly through deamidation and isomerisation (unpublished data), and thus, is suitable for the purpose of this investigation. In this study, glycerol concentrations between 0 and 90% *v/v* were investigated, which includes the range utilised when glycerol is used as an excipient in injectables i.e., 1.6–70% *w*/*v* [30].

## 2. Materials and Methods

### 2.1. Materials and Reagents

The GA-Z protein was supplied by Swedish Orphan Biovitrum AB (Stockholm, Sweden) at a stock concentration of 94 mg/mL, in a 25 mM sodium phosphate buffer (Merck Millipore, Burlington, MA, USA), 125 mM NaCl (VWR, Radnor, PA, USA) at pH 7.0, and stored at −80 °C before use. GA-Z has a molecular weight of 11.9 kDa and an isoelectric point of approximately 4.5. Acetonitrile (HPLC graded, 99.9%), trifluoroacetic acid (HPLC graded, > 99%), and sodium phosphate monobasic (H_2_NaPO_4_, ≥ 99 %) were purchased from Sigma-Aldrich (St. Louis, MO, USA). Sodium phosphate dibasic dihydrate (Na_2_HPO_4_•2H_2_O, 99–102%), sodium phosphate dibasic monohydrate (NaH_2_PO_4_•H_2_O, 98.5–100.5%), and sodium chloride (NaCl, 99.5%) were supplied by Merck (NJ, USA). Sodium chloride (100%), glycerol (99.5%), and formic acid (LC-MS graded, 99%) were purchased from VWR (PA, USA). Acetonitrile (LC-MS graded, 99.9%) was purchased from Honeywell (Charlotte, NC, USA). Glycerol-d8 (98%) was purchased from Cambridge Isotope Laboratories, Inc. (Tewksbury, MA, USA). D_2_O (99.8%) was supplied by ARMAR GmbH (Leipzig, Germany). Sodium azide (NaN_3_, 99%) was supplied by Arcos Organics (Morris Plains, NJ, USA). Water used for samples, mobile phases, and the buffer was of Milli-Q grade (Grad 1, Merck Millipore). Importantly, the presence of impurities in glycerol must be avoided, as they may prompt degradation of the proteins [31].

### 2.2. Sample Preparation and Stability Study

Samples in the stability study contained 0–90% *v*/*v* glycerol (VWR), 4, 6, or 9 mg/mL of GA-Z, 25 mM of sodium phosphate buffer (Merck Millipore), and 125 mM of NaCl (VWR) at pH 7.0. Milli-Q water (Merck Millipore) was used in all buffers. Duplicates were made of all samples. The samples were incubated at 37 °C for 0–41 days. To prevent bacterial growth in the samples during the incubation, the aqueous phosphate buffer saline (PBS) and glycerol were autoclaved before use and all samples were prepared under sterile conditions. After incubation, the samples were stored at −80 °C before further analysis.

### 2.3. Water Activity

The water activity of samples containing 9 mg/mL GA-Z and 0–90% *v*/*v* glycerol was measured using the system AquaLab (Decagon Devices, Pullman, WA, USA), at 20 °C. Before measurements, the system was calibrated with pure water (a_w_ = 1) and with two AquaLab reference samples of water activity 0.250 and 0.760 (Decagon Devices, WA, USA). All measurements were performed in triplicate.

### 2.4. Liquid Chromatography

Liquid chromatography was performed to measure the rate of chemical degradation. Samples from the stability study were analysed using an Agilent 1260 infinity system equipped with an Agilent 1260 II infinity DAD UV detector, an Agilent 1260 infinity pump, an Agilent 1260 autosampler (Agilent Technologies, Santa Clara, CA, USA), and a BioResolve reversed phase column (Polyphenyl, 450 Å, 2.7 µ, 3 × 150 mm, Waters, Milford, MA, USA). A gradient was run with mobile phase A (0.08% formic acid (VWR) and 0.02% trifluoroacetic acid (Sigma-Aldrich, St. Louis, MO, USA) in Milli-Q water, and mobile phase B (0.08% formic acid (VRW) and 0.02% trifluoroacetic acid (Sigma-Aldrich) in acetonitrile (Sigma-Aldrich). Mobile phase B increased 10–31% in the 0–15 min period, 31–40% in the 15–45 min period, and 40–95% in the 45–50 min period. All samples were analysed at a concentration of 0.33 mg/mL in 10% acetonitrile (Sigma-Aldrich, St. Louis, MO, USA) and Milli-Q water. The flow was set to 1 mL/min, the injection volume to 5 µL, and the column oven temperature to 60 °C. Signal data were collected at two wavelengths, 215 and 280 nm. Samples were kept at 4 °C in the autosampler during the run. The LC chromatograms were analysed using Open LAB CDS software (Agilent Technologies, Santa Clara, CA, USA).

### 2.5. Liquid Chromatography-Mass Spectrometry

Samples incubated for 41 days containing 0–90% *v*/*v* glycerol and 9 mg/mL GA-Z protein were analysed with LC-MS to specifically identify degradation products. For the analysis, an Agilent 1260 II infinity system (Agilent Technologies, Santa Clara, CA, USA) connected to a 6545 Q-TOF LC/MS (Agilent Technologies, Santa Clara, CA, USA) was used. The Agilent 1260 II infinity system was equipped with an Agilent 1260 II infinity autosampler and an Agilent 1260 II infinity pump. The LC method was the same as described under Section 2.4 Liquid Chromatography except that mobile phase B included LC-MS graded acetonitrile (Honeywell). During minutes 3–50, the LC flow was connected to the MS source. MS was run in the positive mode in the mass range of 100–3000 *m*/*z*, with an acquisition rate of 5 spectra s^−1^. The Dual AJS ESI was set to the drying gas temperature of 350 °C, drying gas flow of 12 L/min, sheath gas temperature of 400 °C, sheath gas flow of 12 L/min, and nebulizer pressure of 55 psi. The capillary voltage was 4500 V, nozzle voltage was 2000 V, fragmentor voltage was 175 V, skimmer voltage was 65 V, and octupole RF voltage was 750 V. Data analysis was performed using the software MassHunter (Agilent Technologies, Santa Clara, CA, USA).

### 2.6. Asymmetrical Flow Field-Flow Fractionation

To analyse the quaternary and tertiary structure of GA-Z in the presence of glycerol, asymmetrical flow field-flow fractionation (AF4) was utilised. AF4 was performed with an Eclipse 3+ system (Wyatt Technology, Santa Barbara, CA, USA) coupled to a UV detector operating at 280 nm (UV-975, Jasco Corp., Tokyo, Japan), a differential refractive index (RI) detector (Optilab T-rEX, Wyatt Technology, Santa Barbara, CA, USA), and a multi-angle light-scattering (MALS) detector (DAWN HELEOS II, Wyatt Technology, Santa Barbara, CA, USA). The system was equipped with an Agilent 1200 HPLC pump (Agilent Technologies, Santa Clara, CA, USA) and an Agilent 1100 autosampler (Agilent Technologies, CA, USA). The trapezoidal short separation channel (Wyatt Technology, Santa Barbara, CA, USA) had a tip-to-tip length of 17.3 cm, and inlet and outlet widths of 1.15 and 0.3 cm, respectively. A 350 μm-thick spacer and a regenerated cellulose membrane (Merck Millipore) with a molecular weight cut-off of 10 kDa were used. As a carrier liquid, PBS (pH 7) was used containing 25 mM of sodium phosphate (Merck Millipore), 125 mM of sodium chloride (VWR), 1 w% sodium azide (Arcos Organics), and 0–9% *v/v* glycerol (VWR) in Milli-Q water (Merck Millipore). The detector flow was 1 mL/min, and the cross flow was constant at 3 mL/min. The injection time was 3 min, the injection flow was 0.2 mL/min, and the focusing time was 9 min. A 2 µL volume of a 9 mg/mL GA-Z sample was injected, corresponding to an injected mass of 18 µg. A change in injection volume did not change the retention time of GA-Z, which shows that the channel was not overloaded. The samples contained PBS buffer and 0–9% *v*/*v* glycerol and were taken from the stability study at day 0, described in Section 2.2 Sample Preparation and Stability Study. The analysis of each sample was performed in triplicate. The samples were kept at 20 °C in the autosampler during the run and experiments were performed at 20 °C. The experimental results were analysed using ASTRA 6.1 (Wyatt Technology, Santa Barbara, CA, USA). The dn/dc used for GA-Z was 0.196 mL/g [32]. MALS data were fitted using the Zimm model with a first-order fit to obtain the molecular weight (MW) of GA-Z. The RI signal was used to calculate the concentration of GA-Z used in the MW analysis.

The retention time of a macromolecule in AF4 depends on the hydrodynamic size of the molecules, and the hydrodynamic diameter can be calculated using Equation (1).

(1) $$r_{h}=\frac{V^{0}kT}{w^{2}\pi t^{0}Q_{cross}\eta }t_{r}$$

*V*^0^ is the void volume of the AF4 channel, *k* is the Boltzmann constant, *T* is the absolute temperature, *w* is the thickness of the channel, *t*^0^ is the void time, *Q_cross_* is the cross flow, and *η* is the dynamic viscosity of the carrier liquid. The equation is obtained by combining the AF4 theory [33] and the Stokes–Einstein equation. The void time is obtained as described elsewhere [34] and the channel thickness was determined from the elution time of bovine serum albumin according to the procedure of Magnusson et al. [35].

### 2.7. Small Angle Neutron Scattering

Small-angle neutron-scattering (SANS) experiments were performed on the KWS-2 instrument at the Heinz Maier-Leibnitz Zentrum [36] (MLZ, Garching, Germany) to examine how glycerol changes the conformation of GA-Z. Two detector distances were used (2 and 8 m) and a neutron wavelength of 5 Å. The combined data from these two configurations provided a combined q-range of 0.008–0.45 Å^−1^. Samples placed into 2 mm path length “banjo” quartz cuvettes were loaded in a temperature-controlled sample changer and measured at 25 °C. The raw data were reduced according to the protocol of the beamline accounting for detector efficiency, background noise, sample transmission, and scattering from an empty cell. The solvent contribution was subtracted, and data were reduced to obtain the output 1D files in absolute scattered intensity, I(q) in cm^−1^, vs. momentum transfer, q in Å^−1^.

Samples for SANS measurements were prepared using the following protocol. GA-Z stock solution was dialysed against 25 mM of sodium phosphate buffer (Sigma-Aldrich) and 125 mM of NaCl (Merck) in D_2_O (ARMAR GmbH) at pH 7.4 (corresponding to a dH of 7.0 in D_2_O) [37] using the Slide-A-Lyzer G2 Dialysis Cassette (Thermo Fisher Scientific, Waltham, MA, USA). The dialysis was performed according to the manufacturer’s manual. NanoDrop (Thermo Fisher Scientific, MA, USA) was used to calculate the concentration of the protein after dialysis. The sample was analysed with SANS containing 4, 6, and 9 mg/mL of protein, 25 mM of sodium phosphate buffer (Sigma-Aldrich), 125 mM of NaCl (Merck) at pH 7.4 in D_2_O, and 0–90% *v*/*v* of glycerol-d8 (Cambridge Isotope Laboratories).

The absolute scattering intensity in the low angle for a centrosymmetric, uniform scatterer can be described using Equation (2)

(2) $$I\left(q\right)=\phi V_{particle}{}^{2}{\left(SLD_{particle}-SLD_{solvent}\right)}^{2}P\left(q\right)Sq$$

where *ϕ* is volume fraction and *V_particle_* is the volume of the particle. The intraparticle scattering, which relates to the morphology of the scatterer, is described by *P*(*q*). *S*(*q*) relates to the interparticle scattering and thus accounts for interparticle interactions. As samples were prepared at low concentrations and in saline buffer, electrostatic and steric interactions should be negligible and thus *S*(*q*) = 1. Therefore, the analysis of the data was used to determine the morphology of the scatterer, *P*(*q*).

SANS data were initially evaluated using Indirect Fourier Transform (IFT) [38], implemented in ATSAS GNOM 4.6 [39,40]. IFT is a model-free approach for the real-space analysis of small-angle scattering data. The method calculates the pair-distance distribution function p(r) of a given scatterer as a function of the real space. The p(r) accounts for the size distribution of the particles as a q^2^-weighted histogram of the distances within the scatterer. It is defined by the maximum dimension of the scatterer (D_max_) and the shape of the function, which provides a quantitative interpretation of the morphology and size of the scatterer [41].

Moreover, SANS data were analysed using a model-based approach implemented in SasView 4.2.2 [42]. The shape of GA-Z can be appropriately described using a mathematical model that describes a barbell morphology [43,44]. This model simulates the scattering from two spherical domains of radius r_bell_ linked by a cylinder of radius r_bar_ and length L_bar_. In order to account for anisotropy and shape fluctuations within the globular domains, a Schulz polydispersity function for the domain radius was applied [45]. The function was parametrised with PDI = 0.15 (z = 5.7), Npts = 80, and Ns = 8. Instrument resolution was accounted for by smearing the theoretical models using a Gaussian function at a variable dq/q as determined from the configuration of the instrument.

## 3. Results and Discussion

**Figure 1:**
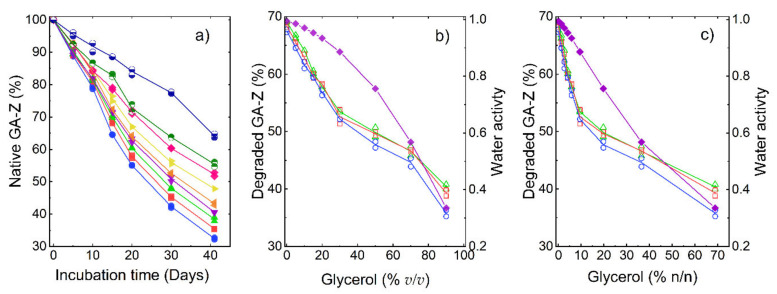
Degradation of GA-Z after incubation at 37 °C as determined by LC-UV. (**a**) Relative amount of remaining native protein vs. incubation time. Samples contained PBS buffer (25 mM, 125 mM NaCl, pH 7.0), 9 mg/mL, and 0% (●), 5% (■), 10% (▲), 15% (▼), 20% (◀), 30% (▶), 50% (◆), 70% (◓), and 90% (◒) *v*/*v* degraded protein and water activitv glycerol. (**b**) Relative amount of degraded protein and water activity (◆) vs. glycerol concentration (% *v*/*v*) after 41 days of incubation. (**c**) Relative amount of degraded protein and water activity (◆) vs. glycerol concentration (% n/n) after 41 days of incubation. (**b**,**c**) samples contained 4 (○), 6 (□), and 9 mg/mL (△) of GA-Z.

The chemical degradation of GA-Z in the solution was determined using LC-UV after incubation at 37 °C. The protein was degraded by deamidation, isomerisation, and hydrolysis (LC-MS data, Figures S1–S3) [46,47], and the total degradation was defined as the relative peak area of native GA-Z left after incubation. The results of the chemical stability study are presented in Figure 1. The addition of glycerol significantly enhanced the chemical stability of GA-Z, as the relative amount of preserved native protein increased with increasing glycerol concentration (Figure 1a). At 0% *v*/*v* glycerol, 32% of native GA-Z remained after 41 days of incubation at 37 °C. At the highest investigated concentration of glycerol, 90% *v*/*v*, the degradation of native GA-Z was strongly decreased, and 64% native GA-Z remained after 41 days at 37 °C. It was also found that the GA-Z concentration had a subtle but distinct impact on the chemical stability of the protein (Figure 1b,c). In the concentration range investigated (4–9 mg/mL), a higher GA-Z concentration resulted in slightly less degradation of the native GA-Z both in the absence and presence of glycerol, where a more pronounced increase in stability is observed at high glycerol concentrations. The chemical degradation of the protein did not induce any detectable aggregation, as seen by AF4-UV-MALS-RI. Therefore, the decrease in native GA-Z is solely attributed to chemical degradation reactions.

The main chemical degradation reaction routes are all dependent on the availability of water, and the reactions are therefore severely hindered in its absence [15]. As such, water activity (a_w_) has been proposed as one of the main factors that control the rate of protein degradation [6,10]. In Figure 1b,c, it can be clearly observed that the decrease in degradation caused by glycerol cannot be solely explained by a decrease in a_w_, since it was relatively high (0.89–1) at the glycerol concentrations where the decrease in degradation was highest, i.e., 0–30% *v*/*v* glycerol. At higher glycerol concentrations (>30% *v*/*v*), a_w_ decreased more strongly with an increasing glycerol concentration. Therefore, the decrease in chemical degradation of GA-Z at these glycerol concentrations could be more influenced by the lower a_w_. However, protein chemical degradation is closely linked to its structure and peptide backbone mobility [9]. Therefore, factors other than solvent properties, such as a_w_, should impact the chemical degradation rate of GA-Z. Thus, the protein structure was investigated in different solution conditions.

**Figure 2:**
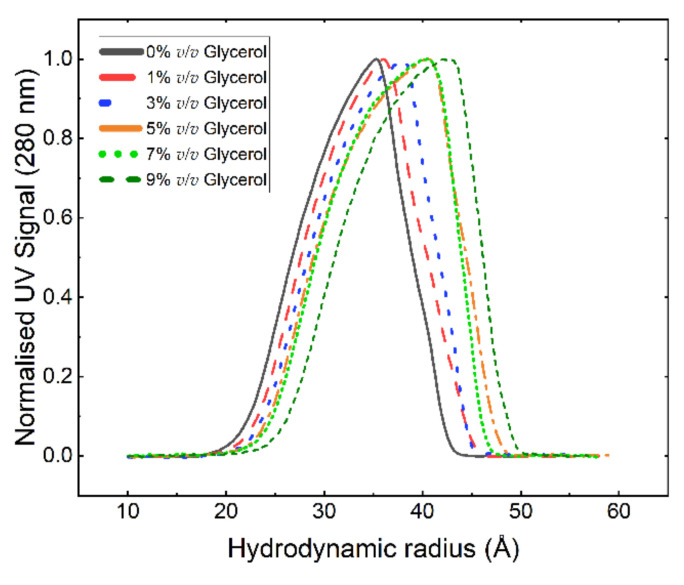
Normalised hydrodynamic radius distributions for GA-Z in PBS buffer (25 mM, 125 mM NaCl, pH 7.0) and 0–9% *v*/*v* glycerol. The recovery in mass, determined via UV signal (280 nm) of the analysed samples, was 83%, 87%, 87%, 96%, 81%, and 73%, respectively. The hydrodynamic radius was determined from AF4-UV elution times (Figure S4) using Equation 1. All analyses were performed in triplicate.

The effect of glycerol on the protein’s tertiary and quaternary structures was investigated using AF4 and SANS. Figure 2 shows the hydrodynamic radius (r_h_) distribution of GA-Z in 0–9% *v*/*v* glycerol determined from AF4-UV elution times (Figure S4). The elution time was corrected for the increase in carrier liquid dynamic viscosity, caused by glycerol addition [48]. The results show that r_h_ increased with an increasing glycerol concentration, manifested by a shift in the distribution maximum and a broadening of the distribution (Figure 2). The molecular weight (MW) of GA-Z obtained with AF4-MALS-RI was determined as 11 ± 0.4 kDa (Figure S4), which corresponds to the MW of GA-Z monomer (11.865 kDa as determined by MS). Upon the addition of glycerol, the MW did not increase over the elution peak, confirming that glycerol did not cause the formation of any (unresolved) oligomers (Figure S4). Therefore, it can be concluded that the change in the r_h_ and, thus, the decrease in chemical degradation reaction rate was not caused by a significant change in the quaternary structure of GA-Z. The r_h_ is relatively large for a low-MW protein like GA-Z and comparable to, for instance, bovine serum albumin with an MW of 66 kDa and an r_h_ of approximately 39 Å [35]. As the MW remained unchanged, the shift in r_h_ with an increasing glycerol concentration must then relate to changes in the protein’s tertiary structure, leading to an increase in size. The unexpectedly high values for r_h_ could be explained by elongated conformations becoming increasingly favoured when glycerol was added. Upon the depletion of glycerol from the GA-Z solution (i.e., dilution with buffer), the glycerol-induced changes in conformation are readily reversible, as shown in Figure S5. Thus, lasting conformational effects, which could influence biological activity, are absent.

**Figure 3:**
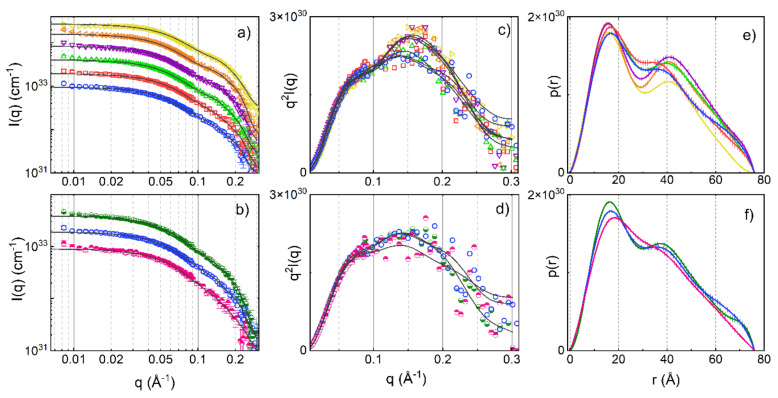
SANS data of GA-Z dissolved in deuterated PBS buffer (25 mM, 125 mM NaCl, pH 7.0). (**a**) Scattering curves and (**c**) Kratky plot for samples with 6 mg/mL and 0% (○), 5% (□), 30% (△), 50% (▽), 70% (◁), and 90% (▷) *v*/*v* glycerol-d8. (**b**) Scattering curves and (**d**) Kratky plot for samples with 4 (◓), 6 (○), and 9 (◒) mg/mL, and 0% glycerol-d8. Symbols represent experimental data, and the solid lines correspond to IFT fits. The SANS data were scaled by a factor (×2, ×4, ×8, ×16, etc.) for clarity. (**e**,**f**) p(r) functions from the IFT analysis of SANS data in (**a**,**b**), respectively. Error bars (standard deviation) are shown for all data. Where not visible, the error bars are overlapping with symbols.

To further characterise and understand the conformational changes induced by the addition of glycerol, the structure of GA-Z in the solution was determined using SANS. Figure 3 shows the SANS data and Kratky plots of samples containing 6 mg/mL GA-Z in the buffer with 0–90% *v*/*v* glycerol, and 4, 6, and 9 mg/mL GA-Z in the buffer without glycerol. The scattering intensities increased with higher concentrations of glycerol and protein due to the higher scattering length density of glycerol compared to water and the higher volume fraction of the protein, respectively. The data were therefore normalised to the contrast factor, i.e., the square of the scattering contrast (ΔSLD)^2^, and to the volume fraction of the protein, according to Equation (2). The scattering curves in Figure 3a,b show a characteristic shape of two oscillations at approximately 0.05 and 0.18 Å^−1^. This scattering pattern is in good agreement with what is expected from the anisotropic morphology of GA-Z i.e., two globular domains linked by a peptide chain [49], and conforms with what has been previously reported for the solution structure of structurally similar proteins [50]. In the scattering curve of anisotropic proteins, the most frequently occurring distances of the scatterer display themselves as maxima or shoulders [49]. The first shoulder (approximately 0.18 Å^−1^) corresponds to the scattering of the individual domains and the second shoulder (approximately 0.05 Å^−1^) correlates with the distance between the two domains [43,49]. The oscillations are more easily observed in the Kratky plots (Figure 3c,d) where they appear as a shoulder and a second peak.

In Figure 3e,f, the calculated p(r) functions of the analysed samples are shown. The plots contain two distinct peaks around 16 and 40 Å, and a shoulder at high q (approximately 70 Å). The first peak around 16 Å arises from the scattering within each of the two domains [43,49] and the position of the first peak relates to the dimension (radius) of the domains. The individual domains are approximately 15 × 25 Å in size, as judged by the crystal structure of structurally similar proteins [51,52,53,54,55], which agrees with the position of the first peak. The second peak corresponds to distances between the two domains [43,49] and, as such, provides direct information of the protein elongation. The two domains connected by the linker are not locked in a fixed position and the shape of GA-Z cannot be interpreted as a rigid body according to small-angle scattering data modelling [32]. The domains are constantly moving around the linker and the protein can therefore obtain multiple conformations. The distances between the domains, represented by the second peak can thus be related to the ensemble of possible conformations due to fluctuations in the protein tertiary structure.

**Figure 4:**
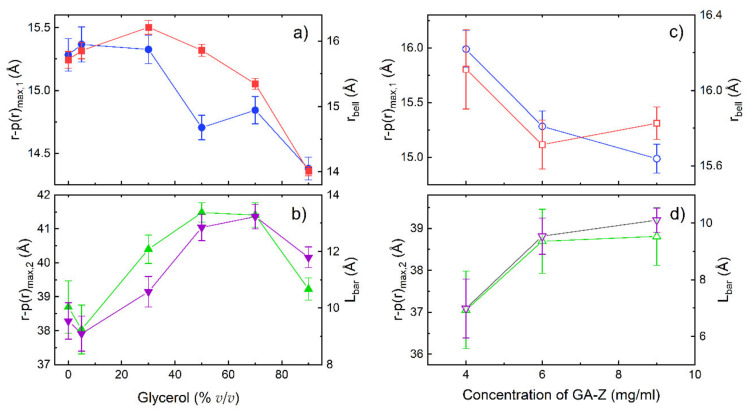
SANS data from deconvoluted p(r) functions and barbell model analysis. (**a**,**b**) r-p(r)_max,1_ (●), r_bell_ (■), r-p(r)_max,2_ (▲), and L_bar_ (▼) vs. glycerol-d8 concentration, (**c**,**d**) r-p(r)_max,1_ (○), r_bell_ (□), r-p(r)_max,2_ (△), and L_bar_ (▽) vs. GA-Z concentration. The r values at the first and second peak maxima of the p(r) functions, r-p(r)_max,1_, and r-p(r)_max,2_, were obtained through deconvolution (Figure S7). The radius of the bell (r_bell_) and the length of the linker (L_bar_) were obtained through the barbell model analysis. Error bars (standard deviation) to the error-weighed fitting of p(r) are shown for all data.

To extract quantitative information from the changes in the shape of the p(r) curves, the curves were deconvoluted using two Gaussian functions and a flat baseline (Figure S7), and the r values of the two peak maxima were extracted. The results are shown in Figure 4. When glycerol was added, changes in the position of the two peaks in the p(r) functions were observed. The r values at the first peak maxima, r-p(r)_max,1_ decreased with an increased glycerol concentration (Figure 4a). In order to rationalise the structural changes in protein structure, data were also analysed using a model-based approach that calculates the scattering from a barbell structure as described in the Materials and Methods section. As an accurate determination of the radius of the linker is beyond the resolution of the SANS measurements, this parameter was fixed to the average radius of the possible conformations of the peptide linker (3.8 Å). This value was determined from the molecular structure of the peptide linker and changes in the value (±1 Å) were not seen to affect the results presented here. The radius of the bell, r_bell_, in the barbell model also decreased with increased glycerol concentration (Figure 4a). The lower r-p(r)_max,1_ and r_bell_ indicate that the protein domains became slightly more compact with increasing glycerol concentration.

The r values at the second peak maximum, r-p(r)_max,2_ also varied with glycerol concentration and significantly increased up to 50% *v*/*v* glycerol (Figure 4c). The barbell model suggests that the length of the linker (L_bar_) increased with glycerol concentration in a similar manner to the increase in r-p(r)_max,2_. Furthermore, the Kratky plot in Figure 3c shows that peaks corresponding to the scattering between the domains shift to higher q with increased glycerol concentration. This shows that protein conformations where the domains are more separated from each other were favoured (i.e., elongated conformations), which is in agreement with the increase in size observed with AF4-UV (Figure 2). Similar trends in conformational changes are seen with varying GA-Z concentrations (Figure 3f and Figure 4c,d), although the magnitude of these changes was smaller.

Glycerol is often added to protein solutions in order to hinder aggregation and denaturation [12]. In this study, the impact of glycerol on chemical degradation and conformation of the pharmaceutical protein GA-Z has been investigated. The results reveal that glycerol changes the tertiary structure of GA-Z and hindered the chemical degradation reactions deamidation, isomerisation, and hydrolysis. Previous studies have shown for relatively short peptides that deamidation decreases with an increased glycerol concentration [6,10]. This protective effect was attributed to the decreased dielectric strength of the solvent at higher glycerol concentrations. Hence, dielectric strength could also play a role in the decrease in degradation in the current investigation.

**Figure 5:**
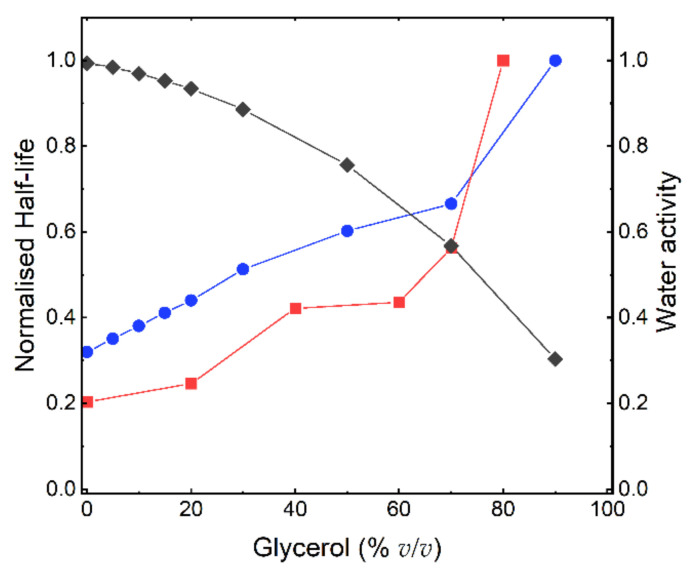
Calculated half-life of GA-Z (●), half-life of peptide-Val-Tyr-Pro-Asn-Gly-Ala (■) from the study by Brennan et al. [10], and water activity (◆) vs. glycerol concentration. The half-life of GA-Z was determined using the data shown in Figure 1 and the calculations are shown in Figure S6.

Figure 5 shows a comparison of the effect of glycerol on the change in degradation rate between GA-Z and a peptide from the study by Brennan et al. [10] plotted as half-life vs. glycerol concentration. When compared to the peptide, the half-life of GA-Z increased more significantly with an increasing glycerol concentration between 0 and 20% *v*/*v*, with a three-times-higher increase in half-life for GA-Z than the peptide (GA-Z = 0.0062, peptide = 0.0021). Unlike GA-Z, the short peptide most likely does not fold into specific three-dimensional conformations. Therefore, the inhibition of chemical degradation of the peptide is strictly related to the primary structure of the peptide and solvent properties, such as water activity and dielectric strength. Between 0 and 30% *v/v* glycerol, the water activity was still high (Figure 1b), and it is thus not surprising that the effect of glycerol on the stability of the peptide was relatively low in this region. However, GA-Z shows a high increase in chemical stability at these glycerol concentrations. This is hypothesised to arise from the changes in GA-Z conformation induced by the presence of glycerol. When glycerol is added to the system, conformational changes are observed in the results both from AF4 (Figure 2) and SANS (Figure 3 and Figure 4) where the addition of glycerol favoured elongated conformations of the whole protein, as well as a compaction of the two protein globular domains. Hence, the results clearly show that the glycerol-induced conformational changes already increased the chemical stability of GA-Z at relatively low glycerol concentrations.

The compaction of the domains agrees with previous studies showing that glycerol decreases the apparent specific volume and compressibility of proteins at up to 40% *v*/*v* glycerol [18,23]. Similarly, it has been shown through molecular dynamics simulations that the compressibility of lysozyme in 60% *v*/*v* glycerol was decreased compared to lysozyme in pure water [19]. In the case of GA-Z, a gradually increasing compaction of the globular domains was observed when increasing the glycerol concentration, up to 90% *v*/*v* glycerol. The role of conformation for deamidation and isomerisation rates has been shown in several studies [9,14,15]. Compaction of the domains decreases the mobility of the peptide backbone, which therefore may contribute to the lower chemical degradation rate of GA-Z, according to Clarke’s conjecture [9]. The apparent elongation of the linker could also be related to a stiffer peptide backbone, where the mobility of the linker decreases upon the addition of glycerol. This will also influence the chemical degradation if the reactions occur in this section of the protein, which could explain the decrease in chemical degradation at 0–50% *v*/*v* glycerol. Glycerol-induced compaction may also decrease the water accessibility as water molecules are released from the protein structure [19,23], and lower water accessibility decreases deamidation [15].

The changes in the conformation of the protein may result from the preferential exclusion of glycerol. The decrease in protein apparent partial specific volume, caused by glycerol, is attributed to the energy cost of the exclusion of glycerol from the surface of the proteins. To reduce the energy cost, a protein decreases its contact area by decreasing its volume [18,23]. Experimental data show that glycerol is preferentially excluded from the surface of proteins at the glycerol concentrations of 10–40% *v*/*v* [18,26,27]. Hirari et al. [12] analysed the solvation layer of myoglobin at 0–75% *v*/*v* glycerol and showed that glycerol was excluded up to 30–40% *v*/*v* glycerol. This result is in alignment with the result of Gekko et al. [18], showing that the preferential hydration of proteins is constant up to 40% v/v. Above 40% *v*/*v*, glycerol began entering the protein solvation shell and at 75% *v*/*v* the protein was neutral solvated by glycerol [12]. In the case of GA-Z, at ≥30% *v*/*v* glycerol there is a drastic change in the profile of the degradation vs. glycerol plot (Figure 1b,c) and the decrease in degradation rate is lower >30% *v*/*v* glycerol. The drastic change in degradation rate at ≥30% *v*/*v* (approximately 10% n/n) glycerol may therefore be either a result of this observed change in solvation layer or a change in conformation caused by the change in the solvation shell. However, the chemical degradation of the protein depends on several factors such as the position of the degraded residue in the protein, water accessibility, and the type of degradation reaction. For a more comprehensive understanding of the effect of these factors, a detailed investigation of the degradation routes is needed.

The conformational changes of GA-Z induced by glycerol seen in SANS are also observed when increasing the protein concentration at 0% *v*/*v* glycerol, although these changes were smaller than those in the presence of glycerol (Figure 3f and Figure 4c,d). As such, an increase in the concentration of GA-Z is connected to a change in protein conformation and a decrease in the chemical degradation reaction rates even though there are no changes in solvent properties. This further underlines the importance of the conformational changes for the decrease in chemical degradation reactions.

## 4. Conclusions

Several studies have shown that glycerol can increase the physical stability of protein by lowering the rate of aggregation and denaturation. This study reveals that glycerol also has the ability to strongly increase the chemical stability of GA-Z by lowering the rates of deamidation, isomerisation, and hydrolysis. Importantly, it is shown that the protective effect of glycerol is not solely governed by the properties of the solvent (i.e., water activity and dielectric strength). At lower glycerol concentrations, the decrease in degradation is mostly due to a glycerol-induced change in the tertiary structure of the protein, while the quaternary structure remains unchanged. The conformational changes shown by AF4 and SANS agree with previous studies examining the effect of glycerol on protein structure. Glycerol is shown here to compress the domains of GA-Z and favour elongated overall conformations.

Interestingly, an increase in the GA-Z concentration also slightly lowered the chemical degradation reaction rates and showed similar trends in conformational changes of the protein. This further supports the conclusion that the chemical degradation reaction rates depend on the conformation of the protein. It also shows that the protein conformation can be influenced by changing various system properties, such as the concentration of protein and excipients. These results open a new perspective on the relationship between protein chemical degradation and conformation, where a more chemically stable protein state can be induced by different formulation aspects. The results presented for the model protein will also help to develop better methods for the formulation and preservation of proteins of technological and pharmaceutical interest.

## Supplementary Materials

The following are available online at https://www.mdpi.com/article/10.3390/pharmaceutics13111853/s1, Figure S1: UV chromatograms of GA-Z obtained with LC-UV, Figure S2: Total Ion Chromatogram of degraded GA-Z after 41 days of incubation at 37 °C obtained with LC-MS, Figure S3: MS data of degraded GA-Z after 41 days of incubation at 37 °C obtained with LC-MS, Figure S4: Relative UV AF4 fractogram and molecular weight of GA-Z in PBS buffer and 0–9% v/v glycerol, Figure S5: Relative UV AF4 fractogram of GA-Z in PBS buffer, Figure S6: Calculation of half-life of GA-Z after incubation at 37 °C using logarithmic curve fitting. Figure S7: Deconvolution of p(r) functions using 2 convoluted Gaussian functions and a flat baseline set at p(r) = 0.
