# Supplementary material for: The Impact of Glycerol on an Affibody Conformation and Its Correlation to Chemical Degradation
Source: Pharmaceutics. 2021 Nov 3;13(11):1853. doi: 10.3390/pharmaceutics13111853 (PMC8618440; doi:10.3390/pharmaceutics13111853)
Supplement: Supplementary file 1 [file pharmaceutics-13-01853-s001.zip › pharmaceutics-1409027-supplementary.pdf]

pharmaceutics

Supplementary Materials: The Impact of Glycerol on an Affibody Conformation and Its Correlation to Chemical Degradation

Ingrid Ramm [1,*], Adrian Sanchez-Fernandez [1], Jaeyeong Choi [1], Christian Lang [2], Jonas Fransson [3,+], Herje Schagerlöf [4], Marie Wahlgren [1] and Lars Nilsson [1]

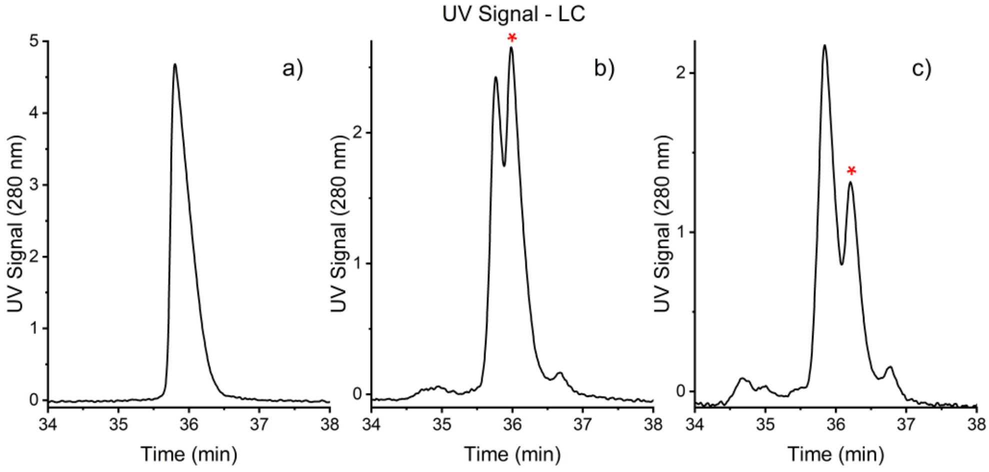

Figure S1. UV chromatograms of GA-Z obtained with LC-UV, showing the degradation of GA-Z after (**a**) 0 days of incubation (native), (**b**) 20 days of incubation at 37 °C, and (**c**) 41 days of incubation at 37 °C. The samples contained PBS buffer (25 mM, 125 mM NaCl, pH 7.0), 9 mg/mL GA-Z, and 0% *v*/*v* glycerol. The peak containing native GA-Z is marked with asterisk (*) and is decreasing in intensity after degradation.

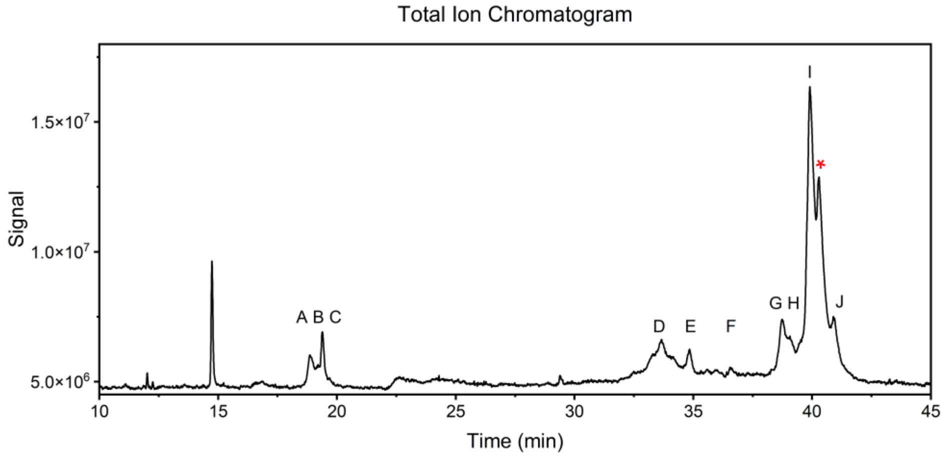

Figure S2. Total Ion Chromatogram of degraded GA-Z after 41 days of incubation at 37 °C obtained with LC-MS. The sample contained PBS buffer (25 mM, 125 mM NaCl, pH 7.0), 9 mg/mL GA-Z, and 0% *v*/*v* glycerol. GA-Z was degraded by deamidation, isomerisation, and hydrolysis. Peaks corresponding to degradation products are marked with the letters, A-J, and their corresponding MS spectra are seen in Figure S3. The peak containing native GA-Z is marked with asterisk (*).

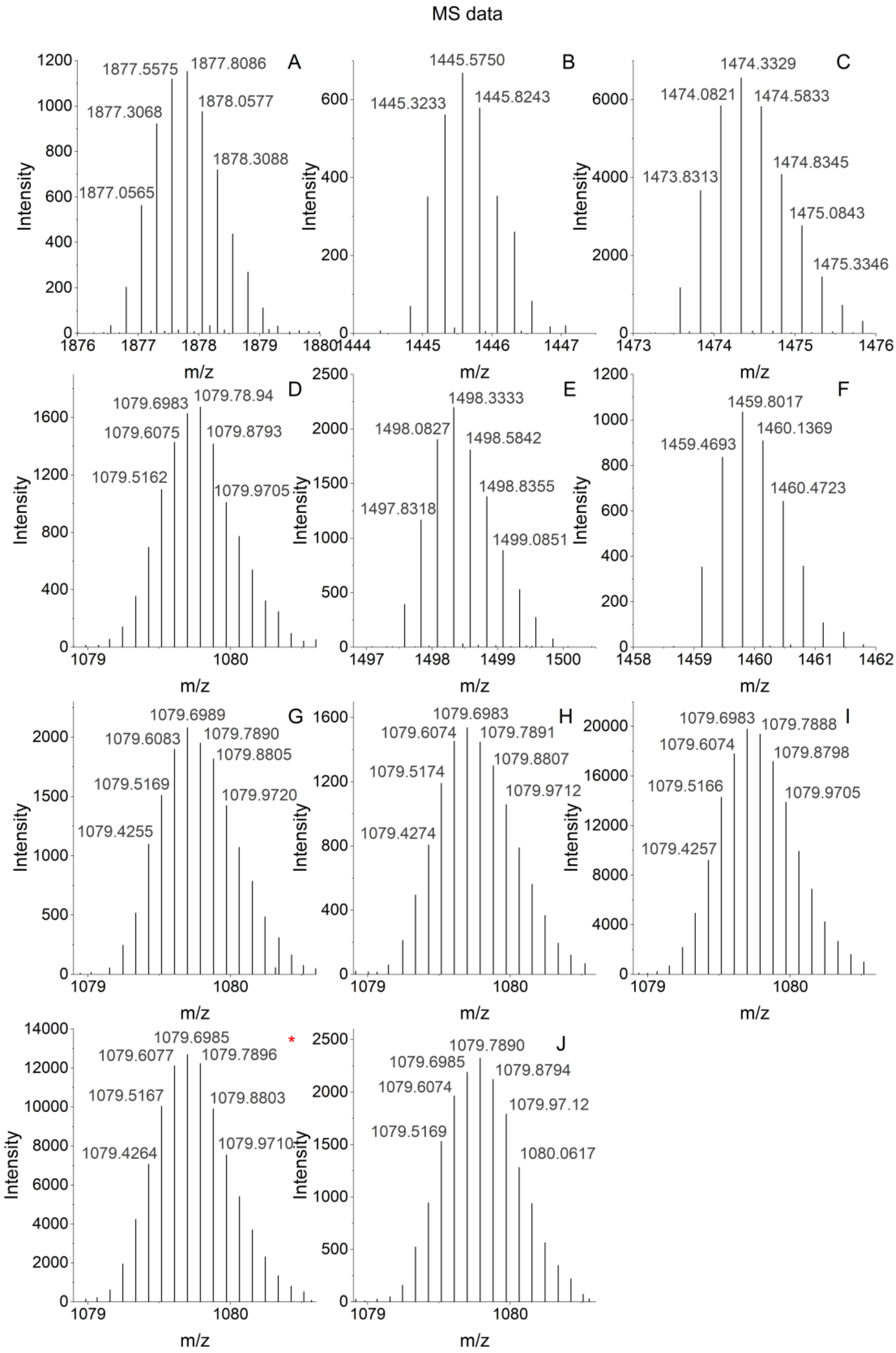

Figure S3. MS data of degraded GA-Z after 41 days of incubation at 37 °C obtained with LC-MS. The sample contained PBS buffer (25 mM, 125 mM NaCl, pH 7.0), 9 mg/mL GA-Z, and 0% *v*/*v* glycerol. Peak * is the native GA-Z protein having a mass of 11 865 Da. Peak A, B, E, and F are hydrolysis products with the masses 7506.87 Da, 5778.01 Da, 5893.04 Da, and 5989.04 Da, respectively, corresponding to fragments of the protein. Peak D and J are deamidation products with a mass of 11.866

Da. Deamidation is manifested by an increase in mass of 1 Da [46,47]. Peak G, H, and I are isomerisation peaks with a mass of 11.865 Da. In LC isomerisation products changes their retention time while their mass remains unchanged [46,47].

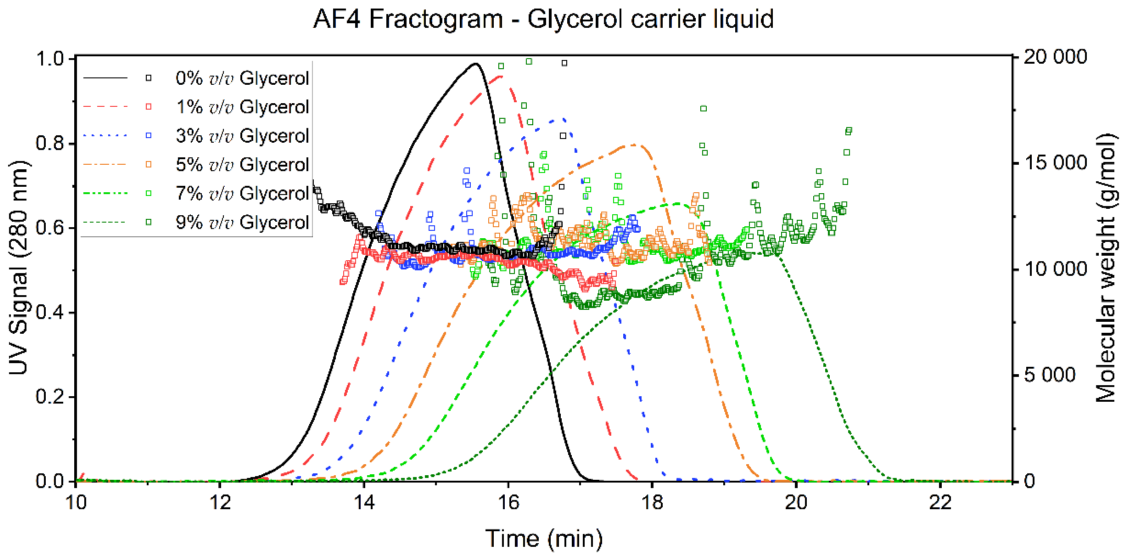

Figure S4. Relative UV AF4 fractogram and molecular weight (MW) of GA-Z in PBS buffer (25 mM, 125 mM NaCl, pH 7.0) and 0–9% *v*/*v* glycerol. MW was obtained with AF4-MALS-RI. Samples contained 9 mg/mL GA-Z, and 0, 1, 3, 5, 7, and 9% *v*/*v* glycerol.

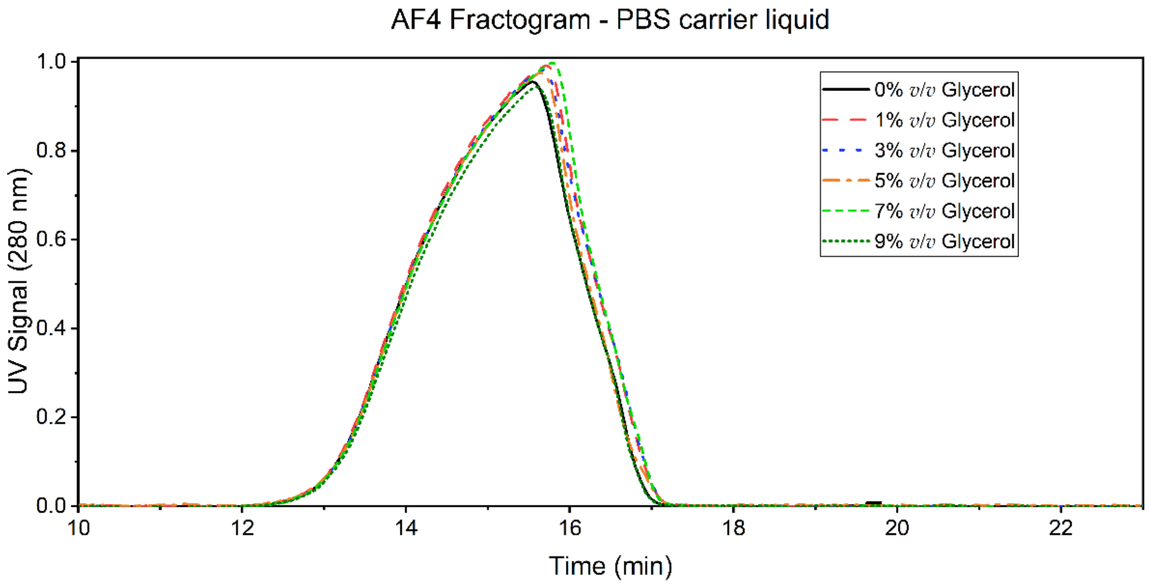

Figure S5. Relative UV AF4 fractogram of GA-Z in PBS buffer (25 mM, 125 mM NaCl, pH 7.0). Samples contained 9 mg/mL GA-Z, and 0, 1, 3, 5, 7, and 9% *v*/*v* glycerol.

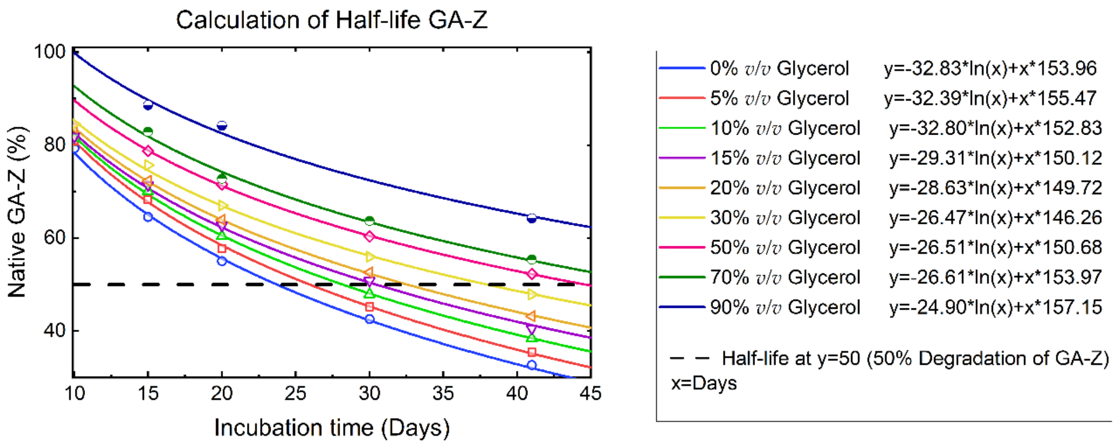

Figure S6. Calculation of half-life of GA-Z after incubation at 37 °C using logarithmic curve fitting. Samples contained 9 mg/ml, PBS buffer (25 mM, 125 mM NaCl, pH 7.0), and 0% (○), 5% (□), 10% (△), 15% (▽), 20% (◁), 30% (▷), 50% (◇), 70% (◓), and 90% (◒) *v*/*v* glycerol. Symbols represent experimental data (Figure 1) and solid lines are the fitted equations shown in the figure.

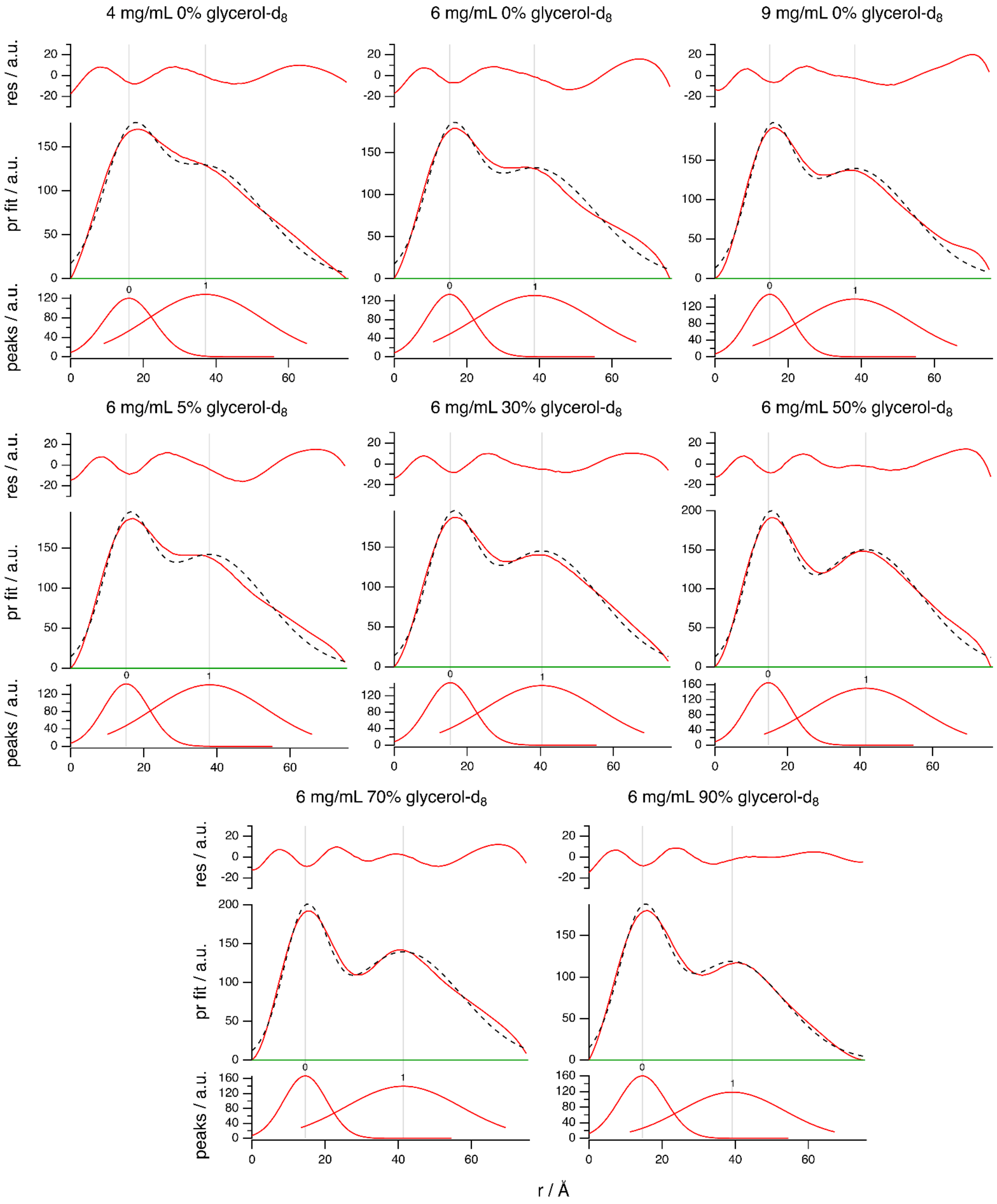

Figure S7. Deconvolution of p(r) functions (Figure 3e and f) using 2 convoluted Gaussian functions and a flat baseline set at p(r)=0.

References

46. .Yang, H.; Zubarev, R.A. Mass spectrometric analysis of asparagine deamidation and aspartate isomerization in polypeptides. Electrophoresis 2010, 31, 1764–1772. https://doi.org/10.1002/elps.201000027.
47. Kori, Y.; Patel, R.; Neill, A.; Liu, H. A conventional procedure to reduce Asn deamidation artifacts during trypsin peptide map-ping. J. Chromatogr. B 2016, 1009–1010, 107–113. https://doi.org/10.1016/j.jchromb.2015.12.009.
